# Comparison of Models to Predict Mechanical Properties of FR-AM Composites and a Fractographical Study

**DOI:** 10.3390/polym14173546

**Published:** 2022-08-29

**Authors:** Juan Leon-Becerra, Octavio Andrés González-Estrada, Heller Sánchez-Acevedo

**Affiliations:** Research Group in Energy and Environment GIEMA, School of Mechanical Engineering, Universidad Industrial de Santander, Bucaramanga 680002, Colombia

**Keywords:** additive manufacturing, thermoplastic composites, machine learning, micromechanics, fractographic analysis

## Abstract

Continuous fiber-reinforced additive manufacturing (cFRAM) composites improve the mechanical properties of polymer components. Given the recent interest in their mechanical performance and failure mechanisms, this work aims to describe the principal failure mechanisms and compare the prediction capabilities for the mechanical properties, stiffness constants, and strength of cFRAM using two distinct predictive models. This work presents experimental tensile tests of continuous carbon fiber AM composites varying their reinforced fraction, printing direction, and fiber angle. In the first predictive model, a micromechanical-based model for stiffness and strength predicts their macroscopic response. In the second part, data-driven models using different machine learning algorithms for regression are trained to predict stiffness and strength based on critical parameters. Both models are assessed regarding their accuracy, ease of implementation, and generalization capabilities. Moreover, microstructural images are used for a qualitative evaluation of the parameters and their influence on the macroscopic response and failure surface topology. Finally, we conclude that although predicting the mechanical properties of cFRAM is a complex task, it can be carried on a Gaussian process regression and a micromechanical model, with good accuracy generalized onto different process parameters specimens.

## 1. Introduction

Additive manufacturing (AM) technologies create an object from a 3D CAD model by adding material layer to layer from information provided by a slicing software, thus, allowing for the manufacture of high complexity parts with minor or no further postprocessing [1]. Additionally, AM materials range continues to grow, from polymers to metals and ceramics [2,3]. Due to their low fusion temperature, one of the first AM materials segments was polymers, particularly thermoplastics. Some of the thermoplastic AM processes are fused filament fabrication (FFF), laminated object manufacturing (LOM), and stereolithography (SLA). Nonetheless, they have the disadvantage of producing not functional or stiff parts, mainly for prototyping but not for structural applications. A solution for this issue are fiber-reinforced additive manufacturing (FRAM) composites, which can improve the material properties of polymeric AM by increasing the stiffness and strength [4]. Nowadays, FRAM applications are in manufacturing fixture tooling, mold equipment, biomedicine, and functional prototyping [5]. Fundamentally, FRAM parts can be of two types: short fiber-reinforced AM composites or long fiber-reinforced, also called continuous-fiber composites. Short fibers are usually shorter than 200 μm. For example, commercially available nylon reinforced with chopped carbon fiber can be printed in consumer desktop 3D printers, such as Prusa i3. In contrast, the continuous fiber-reinforced fiber length goes from 30 mm to even meters, depending on the layer configuration, and requires more specialized and costly equipment [6].

Early research works were focused on determining static mechanical properties of FRAM, such as the stiffness, strength, and failure behavior of different structures subjected to different load types, such as impact, flexural, tension, compression, and interlayer strength [7,8,9]. FRAM significantly improves the stiffness and strength of the raw polymer material, sometimes by a factor of three [10], depending on the fiber type and its configuration. As with traditional composite manufacturing, the load type affects the failure mechanisms [11]. For example, the tension in fiber longitudinal direction is characterized by fiber rupture, while tension in and out-of-plane direction usually causes interlayer debonding. León B. et al. [12] perform a state-of-the-art review of damage and failure mechanisms for continuous fiber-reinforced AM. Diaz-Rodriguez et al. [13] critically review the mechanical properties of FRAM in which the high range of mechanical properties and process parameters are analyzed, revealing that the scattering of the mechanical properties is inherent to manufacturing process parameters, constituent material properties, and environmental conditions. Justo et al. [14] have shown that stiffness and strength depend on the process parameters, such as print direction, type of fiber, layer thickness, and volumetric fiber fraction. The process parameters can be tuned to satisfy or optimize a given mechanical property, as Ahmed et al. [15] show for the optimal variable to enhance the interfacial bond strength.

Given the significant number of possible variations and the unmanageable number of experiments it will take to characterize them fully, researchers are looking for models that accurately predict the mechanical properties and failure behavior of FRAM. We can find models considering a physical interpretation of the stress state [16], numerical experiments [17,18], or raw experimental data for data-driven models [19]. In addition, some researchers are interested in knowing the physical and micromechanical composition of continuous-FRAM for a better physical description of the 3D printed material. One of the critical microstructural descriptors is the volumetric fiber fraction of as-received filament, which is obtained through different methods. Thermogravimetric analysis (TGA) gives the volumetric fiber fraction and thermal behavior of the composite by increasing the temperature, leaving only the fibers, due to the lower fusion and evaporation point of thermoplastics matrices. Moreover, calcination uses the same principle of separating phases using temperature. Other methods involve using chemical acids for dissolving the thermoplastic [20]. The characterization of the matrix chemical nature can be determined using differential scanning calorimetry (DSC). The methods for characterizing the single fiber separate it from the matrix using chemical solvents, and then subject it to mechanical traction. Other authors acknowledge the effects of process parameters and environment on the FRAM properties, for instance, Chabaud et al. [20] investigate the effect of environmental conditions on the stiffness and strength of the FRAM parts by performing thermogravimetric and image processing analysis, showing that the carbon fiber filament has a volumetric fraction of 35% and the fiberglass filament has 39%. The humidity plays an important role, showing variations of 18% in stiffness or 25% in strength for the longitudinal direction, while other directional properties are more affected than in the fiber direction. At the same time, microscopic and image analysis can be used to determine the void fraction [21]. They can also help determine the fiber size, resin-rich or resin-poor matrix regions, and other defects, which can reach values of 15.1% for continuous carbon fiber polyamide cCF/PA parts and 12.3% for cGF/PA parts.

As more physic and microstructural based models appear, a complete description of the behavior of the FRAM for the full characterization of an orthotropic composite 3D printed lamina is needed for engineering calculations and designs. From a composite materials design perspective, nine elastic constants should be given, implying numerous experiments. A helpful approach requiring fewer experiments is obtaining the individual matrix and fiber’s mechanical properties, and then obtaining the overall properties of the composite lamina. For that task, micromechanics deals with the determination of all of the composite properties based on the properties of the constituents. Research has been done in the microstructural characterization of FRAM and micromechanical analysis. Some authors present the transverse area of the FRAM, employing imaging for measuring the volumetric fiber fraction [21]. Moreover, in the work of Pascual-González et al. [22], an extensive experimental micromechanics characterization of AM manufacturing was performed for the single fiber properties, the fiber distribution and content, and the polymer nature. Micromechanics is also valuable for failure behavior, and failure prediction of additive manufacturing composites is the subject of recent investigations. Dutra et al. [23] developed an expanded Puck and Schürmann (ExPan) interfiber fracture criterion, which considers the semi-brittle nature of the thermoplastic matrix. Moreover, they present the failure envelops of 3D-printed composites.

As the number of parameters on which the FRAM mechanical behavior and failure mechanisms depend grows, the experimental work and even the employment of in silico models, such as finite elements, are not feasible. One tool to resolve the problem is using artificial intelligence and data-driven models to predict the properties or design the FRAM architecture. Although machine learning is becoming a powerful tool to tackle challenging problems, a lot of data are required to train a predictive model. Some authors use deep learning to predict the strength of AM parts, while this approach can be used for FRAM parts. Zhang et al. [24] perform a tensile strength prediction of FFF PLA thermoplastic, considering the temporal aspect of the manufacturing process and the time process variations characterized by inter-layer interactions. The model is constructed using the layerwise process signals (vibration and temperature) as inputs of a long short-term memory LSTM network, a recurrent neural network (RNN). Later, Zhang et al. [25] performed the strength prediction for a composite AM sample. This time, they develop an ensemble of different types of machine learning algorithms, such as least absolute shrinkage and selection operator (lasso), K-nearest neighbors, and support vector machines. The input parameters are process-related parameters, such as fiber layers, polymer layers, fiber rings, and infill patterns. Other deep learning applications optimize fiber paths and reverse engineering AM parts [26]. Artificial neural networks (ANN) are also employed as an optimization algorithm that can enhance the mechanical strength of FFF polymers by selecting the optimum process parameters. A deep-learning algorithm proves highly competitive with other optimization algorithms [27].

For connecting the microstructural characteristics with stiffness, strength, and failure mechanisms, Young et al. [28] perform fractographical analysis in additive manufacturing thermoplastic composites, where microstructural differences are found between ABS and CF-ABS, presenting lower porosity values in the former, probably due to the thermal conductivity enhancement of the carbon fiber. Chadha et al. [29] present fractographical evidence of different breaking fracture modes for different infill patterns, such as brittle fracture in a grid structure and ductile fracture in a honeycomb. Other authors could identify fiber slippage, fiber waviness [30], fiber pull out, and interfacial debonding [31]. Furthermore, a counter-intuitive behavior was evident in the work of Seifans et al. [32], where the strength of the 45° reinforced tensile sample was less than 90° angle. Fractographic analysis tools clarify that as the printer deposits a continuous strand of fiber, there are areas in the turns with fiber at 0°, thus elevating the strength.

Given the numerous works in the mechanical characterization of FRAM and the incremental research interest in modeling mechanical properties and failure, there is a need to evaluate micromechanical and data-driven models on continuous FRAM data for predicting the strength. Therefore, this work aims to fill this knowledge gap and elucidate the best possible path by evaluating their accuracy and ease of implementation. First, the micromechanical models are described based solely on constituent properties for the stiffness calculation and in-situ experimental data and back-calculated parameters for the strength prediction. Then, in the second approach, a deep-learning algorithm describes a series of models to perform the stiffness and strength characterization. After establishing the two methods, we introduce the data obtention and curation methodology and the available data set. Finally, comparison and discussion of the results are made.

## 2. Micromechanical Models

### 2.1. Micromechanics for Predicting Stiffness

One of the first attempts to determine the mechanical response of composite materials was the Voigts and Reuss models, namely the rule of mixtures (ROM) and the inverse rule of mixture (IROM), respectively. Those models can be derived from the assumptions of strain equivalences in the axial lamina direction and stress equivalence in the transverse direction. ROM and IROM are depicted in Equations (2) and (3):

(1) $$V_{f}+V_{m}=1$$

(2) $$P=V_{f}P_{f}+V_{m}P_{m}$$

(3) $$\frac{1}{P}=\frac{V_{m}}{P_{m}}+\frac{V_{f}}{P_{f}}$$

In which $V_{f}$ is the volumetric fiber fraction, $V_{m}$ is the volumetric matrix fraction. The subscripts f represent the fiber fraction property value, m is the matrix fraction property value, and *P* is the calculated composite property. These two models provide upper and lower bounds to the composite behavior.

Despite their simplicity, ROM and IROM help predict the longitudinal, $E_{1}$, and transverse, $E_{2}$, elastic moduli quite well, from the fiber, $E_{f}$, and matrix, $E_{m}$, elastic moduli. Moreover, ROM approximates well the in-plane Poisson’s ratio ($v_{12}$) [33]. Other micromechanical formulations are better suited for properties, such as out-of-plane Poisson’s ratios, shear modulus, and out-of-plane behavior. The cylindrical assemblage model (CAM), shown in Equation (4), proposed by Hashin and Rosen, gives better estimates [33] of the in-plane modulus $G_{12}$.

(4) $$G_{12}=G_{m}\left[\frac{\left(1+V_{f}\right)+\left(1-V_{f}\right)G_{m}/G_{f}}{\left(1-V_{f}\right)+\left(1+V_{f}\right)G_{m}/G_{f}}\right]$$

where $G_{f}$ and $G_{m}$ are the shear moduli of the fiber and matrix. Moreover, it is usual to assume that the fiber shear modulus $G_{13}=G_{12}$. The intralaminar shear modulus, $G_{23}$, was computed with the semi-empirical stress partitioning parameter technique as in Equations (5) and (6).

(5) $$G_{23}=G_{m}\left[\frac{v_{f}+\eta_{4}\left(1-v_{f}\right)}{\eta_{4}\left(1-v_{f}\right)+v_{f}G_{m}/G_{f}}\right]$$

(6) $$\eta_{4}=\frac{3-4v_{f}+G_{m}/G_{f}}{4\left(1-v_{m}\right)}$$

In which $v_{f}$ and $v_{m}$ are the fiber and matrix Poisson’s ratio, respectively. Note that more elaborated methods exist, such as homogenization schemes, which consider the reinforced volumetric fraction and their spatial distribution to estimate the mechanical properties of the whole composite. However, the above micromechanical models were used to seek simplicity yet the goodness of fit.

### 2.2. Micromechanics for Predicting Strength

Predicting the strength of a lamina is a more challenging task than a stiffness prediction, despite some authors proposing simple equations that depend on the properties of the constituents and a back-calculation parameter, thus, requiring some experimental data to perform the adjustment. Current micromechanics equations for predicting strength properties have questionable reliability [34]. Usually, they require apparent properties of the constituents (fiber and matrix) obtained from experimental data and then back-calculated using micromechanics formulas. One formula employed for the obtention of the longitudinal tensile strength $F_{1t}$ is depicted in Equation (7). It relies on the assumption that all fibers have the same strength, represented by the apparent fiber tensile strength $F_{ft}$, this assumption is, to some extent, erroneous as the strength of the fibers presents a Weibull distribution [22]. The second assumption is that both matrix and fibers have a linear behavior up to failure, which is not valid, particularly for a polymeric matrix.

(7) $$F_{1T}=F_{fT}\left[V_{f}+\frac{E_{m}}{E_{f}}\left(1-V_{f}\right)\right]$$

Moreover, the equation assumes that once the fibers break, the matrix cannot sustain the load, and the composite fails, which is valid for almost all commercially available composite laminae. However, this is not true for composites with a meager fiber volume fraction. Thus, Equation (8) should be used in those cases.

(8) $$F_{1T}=F_{mT}\left(1-V_{f}\right)$$

where $F_{mT}$ is related to the matrix tensile strength. For the longitudinal compressive strength $F_{1C}$, a simple equation is given by Barbero [35], here replicated in Equations (9) and (10).

(9) $$F_{1C}=G_{12}{\left(1+4.76\chi \right)}^{-0.69}$$

(10) $$\chi =\frac{G_{12}\alpha_{\sigma }}{F_{6}}$$

In those equations $F_{6}$ represent the in-plane shear strength value, $\alpha_{\sigma }$ is the standard deviation of fiber misalignment, measured experimentally [36] or calculated from (10) in terms of available experimental data for $F_{1C}$. Then, Equation (9) can be used to predict the values of $F_{1C}$ for materials with other properties.

The transverse tensile failure of a unidirectional lamina happens when a transverse crack propagates along the fiber direction, splitting the lamina. Therefore, transverse strength is a fracture mechanics problem. We can use Equation (11) to predict the transverse tensile strength $F_{2T}$ of a unidirectional lamina.

(11) $$F_{2T}=\sqrt{\frac{G_{IC}}{1.12^{2}\pi (t_{t}/4)\;\Lambda_{22}^{0}}}$$

where $G_{IC}$ is the fracture toughness in mode I. The transition thickness $t_{t}$ can be approximated as $t_{t}$ = 0.6 mm, 0.8 mm, for E-glass–epoxy and carbon–epoxy composites, respectively. However, $G_{IC}$ and $t_{t}$ are not well established for FRAM composites. Finally, $\Lambda_{22}^{0}$ is given by (12).

(12) $$\Lambda_{22}^{0}=2\left(\frac{1}{E_{2}}-\frac{\upsilon_{12}^{2}E_{2}^{2}}{E_{1}^{3}}\right)$$

Older empirical formulas derived without considering fracture mechanics are also available in [33]. For the transverse tensile, $F_{2T}$, and compressive, $F_{2C}$, strength, empirical formulas are given in Equations (13) and (14):

(13) $$F_{2T}=F_{mT}C_{v}\left[\left(1-V_{f}^{\frac{1}{3}}\right)\;\;\left(\frac{E_{2}}{E_{m}}\right)\right]$$

(14) $$F_{2C}=F_{mC}C_{v}\left[1+\left(V_{f}-\sqrt{V_{f}}\right)\left(1-\frac{E_{m}}{E_{T}}\right)\right]$$

where the apparent compressive strength of the matrix $F_{mC}$ is back-calculated from experimental data on $F_{2C}$ using (14). $C_{v}$ is included to adjust, with another empirical factor, for the presence of voids, and $E_{T}$ is the transverse modulus of the fiber.

(15) $$C_{v}=1-\sqrt{\frac{4V_{V}}{\pi \left(1-V_{f}\right)}}$$

The in-plane shear strength $F_{6}$ is also a fracture mechanics problem. However, it can be approximated using Equation (16) if more experimental data is available for determining the $F_{mS}$ and $C_{v}$ factors. $G_{A}$ is the axial shear modulus of the fiber, which in the case of isotropic fibers is equal to G.

(16) $$F_{6}=F_{mS}C_{v}\left[1+\left(V_{f}-\sqrt{V_{f}}\right)\left(1-\frac{G_{m}}{G_{A}}\right)\right]$$

The intralaminar shear strength $F_{4}$ is a matrix-dominated property because the shear acts on a plane parallel to the fiber direction. The fibers would have to be sheared off by stress $\tau_{13}$ to produce failure, which is unlikely to happen. Equation (17) depicts the intralaminar shear strength in terms of the transverse compressive strength $F_{2C}$ and the angle of the fracture plane $\alpha_{0}$.

(17) $$F_{4}=F_{2C}\cos\alpha_{0}\;\left(\sin\alpha_{0}+\cos\alpha_{0}\cot2\alpha_{0}\right)$$

## 3. Machine-Learning Architectures

In this work, our interest is in predicting the mechanical properties of AM composites. Therefore, it is a regression problem, a kind of supervised learning. Numerous regression models include simple linear regression models, regression trees, support vector machines, Gaussian process regression (GPR) models, and neural networks [37]. The models were tested in the regression learner app from the machine learning and statistics toolbox of MATLAB software 2021. The data set is partitioned into five cross-validations folds and estimates the accuracy on each fold to avoid overfitting.

The [X] denotes the design matrix, also called the training matrix, data matrix, or input matrix, and contains the complete input dataset. The columns correspond to each point in the feature space (training sample) and the rows to each factor. Therefore, it is, in a general way, a non-square matrix. The number of explanatory variables or factors *p*, is the number of rows of [X], while the number of points *m* is the number of columns. The variable {y} is the output, response, or predictor value. It is a column vector in which each row is associated with the corresponding column or feature vector in the data matrix [X]. {w} are the coefficients of the variables in the model, they are also called weights. {ε} represents the error associated with the model, and {b} is called bias, a constant value.

### 3.1. Decision Trees

Regression trees predict responses to data by following a series of decisions in the tree, from the root (beginning) node down to a leaf. The leaf node contains the response [38].

### 3.2. Linear Regression

Linear regression models describe the relationship between the explanatory variables and the *p* number of factors or features. The matrix [X] of observations is an *m* by *p* matrix, with *m* the total number of data points. The response variable is called $y_{i}$ as each point has an associated output value. In general, a linear regression model can be a model of the form represented in Equation (18).

(18) $$y_{i}=w_{0}+\overset{k}{\underset{k=1}{\sum }}w_{k}f_{k}\left(x_{1i},x_{2i},\ldots ,x_{pi}\right)+\varepsilon_{i}$$

where f( ) is a scalar-valued function of the independent variables $x_{ji}$. The functions f(x) might be in any form, including nonlinear functions and polynomials. The coefficients $w_{k}$ are linear. That is, the response variable, *y*, is a linear function of the coefficients $w_{k}$ [37].

### 3.3. Support Vector Machines

Support vector machines (SVM) is a nonparametric technique relying on kernel functions. Matlab regressor app implements linear ε-insensitive SVM (ε-SVM) regression. The goal is to find a function f(x) that deviates from $y_{n}$ by a value smaller than ε for each training point $x_{i}$, and at the same time, it is as flat as possible. So the path to follow is to find a linear function:

(19) $$\left\{\;f\left(x\right)\right\}={\left\{w\right\}}^{T}\left[X\right]+\left\{b\right\}$$

Find f(x) with the minimal norm value $\left(w^{T}w\right)$ formulating a convex optimization problem that has to be minimized:

(20) $$\;J\left(w\right)=\frac{1}{2}{\left\{w\right\}}^{T}\left\{w\right\}$$

Subject to the residuals being less than ε, that is:

(21) $$\forall n:\left|\left\{{\hat{y}}_{i}\right\}-\left({\left\{w\right\}}^{T}\left[X\right]+\left\{b\right\}\right)\right|\leq \varepsilon$$

where $\hat{y}$ is the predicted output.

### 3.4. Gaussian Process Regression

The available data set is in the form $\left\{\left(x_{i},y_{i}\right);i=1,\;2,\ldots ,n\right\}$, where $x_{i}$ ∈ ℝ^d^ and $y_{i}$ ∈ ℝ are drawn from an unknown distribution. A Gaussian process regression (GPR) model predicts the value of a response variable, given the new input vector and the training data. A linear Gaussian regression model is of the form

(22) $$\left\{\hat{y}\right\}={\left\{w\right\}}^{T}\left[X\right]+\left\{\varepsilon \right\}$$

where $\varepsilon ∼N\left(0,\sigma^{2}\right)$. The error variance $\sigma^{2}$ and the coefficients β are estimated from the data. A GPR model explains the response by introducing latent variables, $f\left(x_{i}\right),\;\;i=1,\;2,\ldots ,n,$ from a Gaussian process (GP), and explicit basis functions *h*. A GP is a set of random variables, such that any finite number of them have a joint Gaussian distribution. If $\left\{f\left(x\right),\;x\in {\mathbb{R}}^{d}\right\}$ is a GP, then given *n* observations $\left\{x_{1},x_{2},\ldots ,x_{n}\right\}$, the joint distribution of the random variables $f\left(x_{1}\right),f\left(x_{2}\right),\ldots ,f(x_{n})$ is Gaussian.

### 3.5. Neural Networks

**Figure 1:**
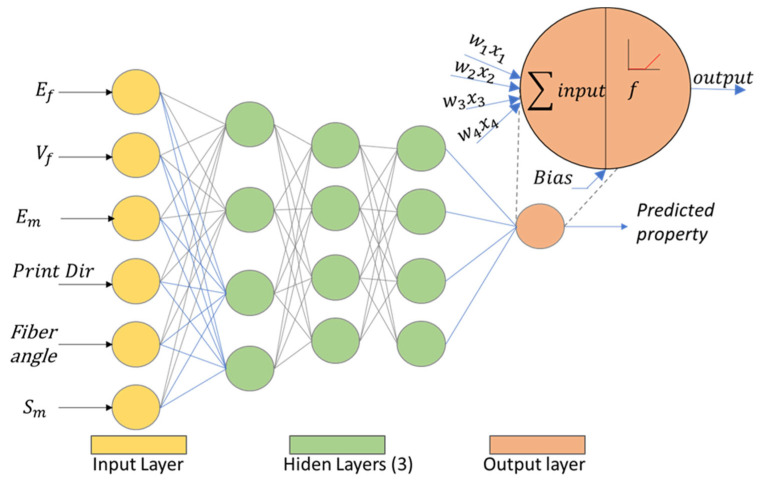
Schematic of the artificial neural network.

Artificial neural networks (ANN) get their name by the resemblance to a neuron, a biological cell, in which usually more than one stimulus enters the neuron, and the response output connects to other neurons in the network. Raw data can be used to train and test an artificial neural network. In this case, the input layer consists of a vector of a set of parameters with dimension *n* = 9, namely: {fiber stiffness, fiber angle, fiber strength, fiber Poissons ratio, matrix stiffness, matrix strength, matrix Poissons ratio, print direction, number of fiber layers}. The output is the stiffness or the tensile strength of such AM parts (Figure 1). The proposed neural network has various hidden layers and a varying number of neurons in each layer, using ReLu for the activation function.

The data set is grouped in two matrices, in which x is the input matrix of dimensions n×m and y is the output matrix of dimension 2×m. They have the form

(23) $$x=\left[x^{\left(1\right)}\;x^{\left(2\right)}\ldots x^{\left(m\right)}\right]$$

The error function employed to evaluate the ANN is the one employed in the gradient descent formulation:

(24) $$ℒ\left(\hat{y},y\right)=-\left(y\log\;y+\left(1-y\right)\log\left(1-\hat{y}\right)\right)$$

While the cost function, which is the parameter to minimize, is given by

(25) $$J\left(w,b\right)=\frac{1}{m}\overset{m}{\underset{i=1}{\sum }}ℒ\left({\hat{y}}^{\left(i\right)},y^{\left(i\right)}\right)$$

## 4. Materials and Methods

Data were gathered from experiments for determining the tensile behavior performed following ASTM D3039, as well as from datasets available in the literature. The raw experimental data are available in Table A1 in Appendix A. The experimental design is presented in Table 1. The experiment design was proposed with three factors, as shown in the columns in Table 1, with various levels at each factor. Although a standardized design of the experiment was not suitable due to the uneven distribution of levels in each factor from manufacturing constraints, at least four specimens per point were tested, accounting for 52 samples.

**Figure 2:**
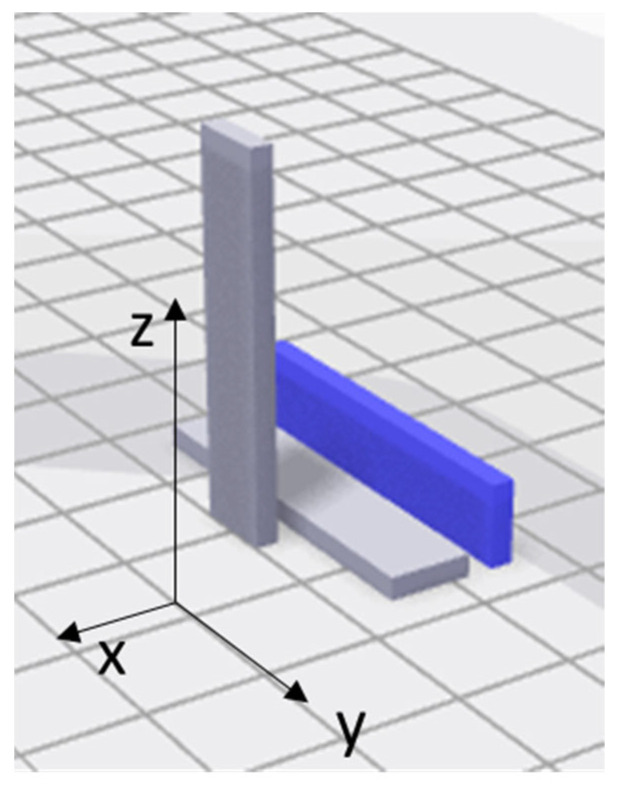
Printing orientations for the tested samples.

MarkTwo desktop printer fabricates the specimens of dimensions 150 mm × 15 mm × 2 mm with a Nylon White matrix and a continuous carbon fiber reinforcement in an aligned arrangement (called “isotropic” in the slicer software Eiger v1.1, Markforged Inc., (Watertown, MA, USA)); Markforged^®^ supplied all the above materials. The total number of layers is 16 in the flat direction and 110 in the on-edge direction, see Figure 2. Each layer has 1.25 mm in thickness. The fiber fraction levels are divided into levels corresponding to 2, 4 and 6 reinforced layers in the flat printing direction; and 28, 42, 55, and 70 reinforced layers in the on-edge printing direction.

**Figure 3:**
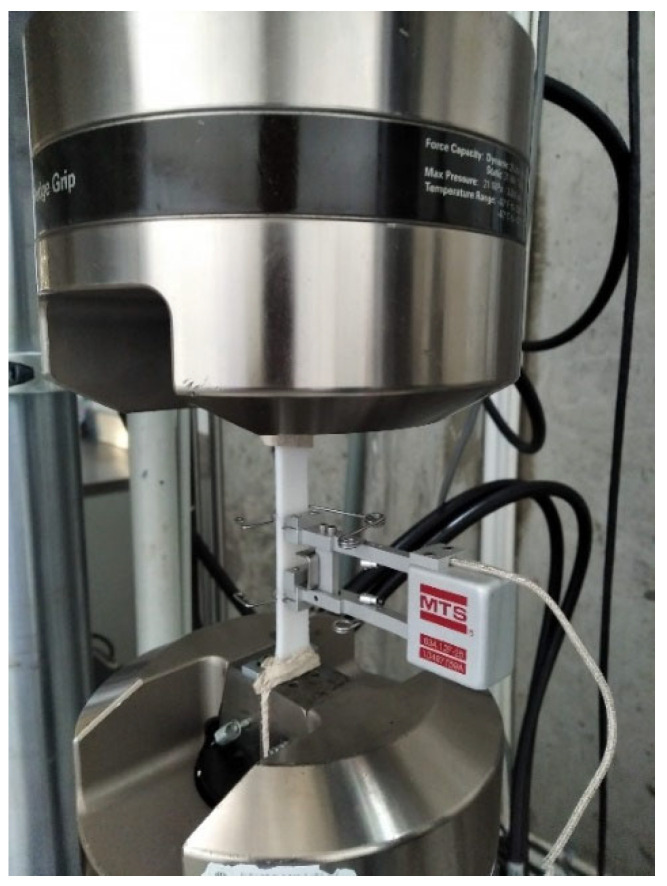
Experimental setup of a tensile specimen.

Samples were visually inspected before testing, and some specimens had minor defects, such as lack of adhesion in local points. However, no general or great-extent defects such as warping, missing layers, or voids were found. Samples are then tested in an MTS Bionix 370.02 with a mechanical MTS 634.12F axial extensometer to accurately determine the elastic response (Figure 3), and the extensometer length is calibrated at 25 mm. The chosen gripping method was cloth, as Pyl et al. [39] show the low variance of the results. The testing test speed was 2 mm/min.

## 5. Results

### 5.1. Overall Results

The average results for Young’s modulus, the maximum stress, and the coefficient of variation (COV) of our experimental data can be observed in Table 2. The data were analyzed through the factorial design of experiments (DOE) analysis and analysis of variance (ANOVA) in the MiniTab software. As a result, the following hypotheses are true: in the flat printing direction, the fiber angle, fiber content, and their interaction affect the average stiffness response (*p*-value 1.32 ×10^−11^); in the on-edge printing direction, the fiber content affects the average strength response (*p*-value 0.34) and stiffness response (*p*-value 0.01). The higher *p*-value of the strength response indicates that the statistical significance of the fiber content in the strength is not established, at least for 45° and 90°.

**Figure 4:**
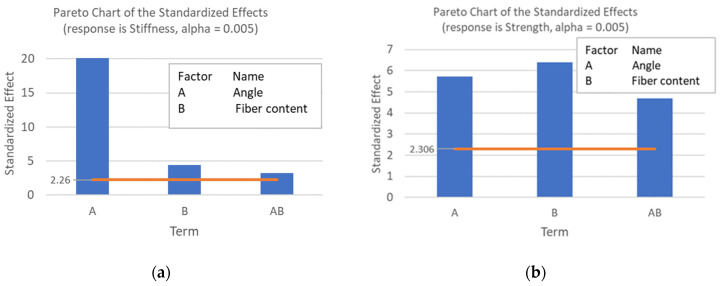
Pareto diagrams for the angle, fiber content, and interaction effects in the (**a**) stiffness and (**b**) strength.

Figure 4 shows the Pareto diagrams of the effects of angle and fiber content on the stiffness and strength, thus, finding that the critical effect in the stiffness is the fiber angle. In contrast, fiber content is more determining for strength than fiber angle. The factor that has the greatest significance in stiffness is the fiber angle, followed by the fiber content and their interaction, being that the fiber angle is almost four times more important than the fiber content. On the other hand, the fiber content is the most relevant factor in the strength, followed by the fiber angle and their interaction. Nonetheless, the importance of the factors is more even between them than in the stiffness case.

**Figure 5:**
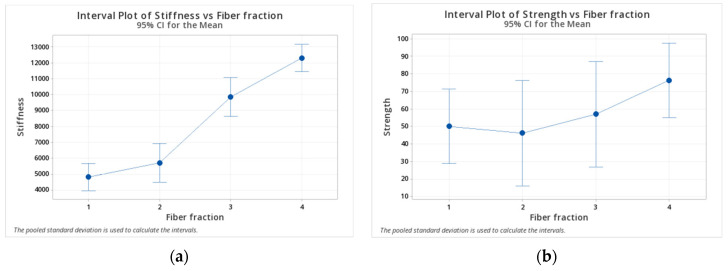
Interval graphs for (**a**) stiffness and (**b**) strength of on-edge specimens.

**Figure 6:**
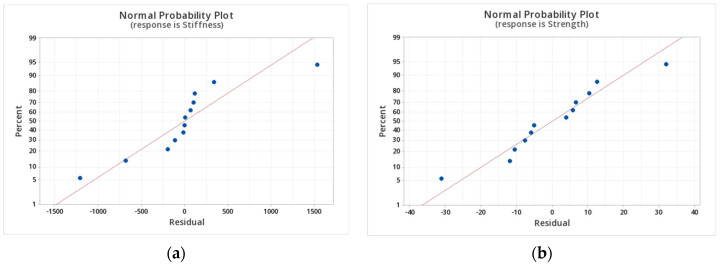
Normal residual plots for (**a**) stiffness and (**b**) strength.

In the case of on-edge printing, the angle is not a factor because it is only possible to print a 0-degree coupon. Figure 5 shows the stiffness and strength interval graphs, while Figure 6 shows the residues plot. A clear increasing tendency of stiffness with fiber fraction is identified in the stiffness plot (Figure 5a). On the contrary, no such tendency is appreciable in the interval plot of strength (Figure 5b). Moreover, given the high dispersions of the values (COV in the order of 25%, see Table 2), the 95% confidence interval is large, thus giving the appearance of one range superposed over another.

Residues plot (Figure 6) can accommodate well into a normal distribution, and this could be seen by the good agreement between the blue experimental points and the red gaussian line distribution. The agreement indicates the validity of the ANOVA analysis and the Pareto charts mentioned before. If that was not the case, a transformation, such as Box–Cox, could be handy. Furthermore, the flat direction is stronger than the on-edge direction, given the same fiber angle and content.

Given the high dispersions of values, the hypothesis that the fiber content affects the average strength response in the on-edge printing direction is not confirmed with 95% confidence, instead only 66% confidence. Thus, more tests should be performed to conclude firmly. Differences and errors could be due to local defects, such as points that do not completely fuse in a portion of the layers, voids in the filament and voids resulting from the printing process, and hygroscopic process in the nylon before and after printing the part [20]. Despite the manufacturing defects, properties dispersion could be seen as regular variations in the fabrication process, and their values do not affect the implications of the results or reproducibility of the tests.

### 5.2. Micromechanics Predictions

First, the comparison of the micromechanical model for stiffness prediction is made based on the analytical equations depicted in (1) to (17) and presented in Table 3, *E*_1_ was computed using Equation (2) and *E*_2_ Equation (3). However, the structure of the 3d printed samples is instead sandwich composites than truly fully composite structures because of the top and bottom layers that are usually printed from raw thermoplastic. In those cases, the volumetric average stiffness (VAS) method [40] is employed to predict the overall behavior.

The values of stiffness and strength for the matrices $E_{m}$, $S_{m}$, and the stiffness of the fibers $E_{f}$ employed were obtained in the corresponding articles, or an informed guess value if not available. The average relative error for the longitudinal modulus is 19%, with typical values between −18% to 70%. There are some outliers with errors as −74% or 91%. Moreover, errors range from −38% to 71% for the transversal modulus with a 48% average error, despite the poor data availability. As the suggested values for the rest of the constants depend on the volumetric fiber fraction, we provide look-up values in in Table 4. The micromechanical properties employed are depicted in the annexed table. For the out-of-plane Poisson’s ratio $v_{23}$, a value between 0.28 and 0.35 for most FRAM is suitable due to the low inference in the structural response. However, a periodic microstructural model could be used to give a more precise approximation.

The obtention of the strength data is cumbersome as various factors can significantly influence the strength of additive manufacturing composites. Different authors enunciated the effect of process parameters on the strength of the overall AM composites and polymers [52,53,54]. Thus, the effect of printing direction in Table 5 shows the strength comparison for our data.

Predicted strength data were obtained using Equation (8) for the 0° reinforced samples and Equation (13) for the 90° reinforced samples. Notice that there is a more significant amount of dispersion between each point. In addition, a counter-intuitive trend exists in the on-edge printing direction, as the 28 layers version resisted a fair amount of stress, reaching close to 42 layers. A possible explanation is the effect of layer-by-layer adhesion on the composite. Thus, conducting to think that this damage mechanism is prevalent with the low volumetric fiber fraction on-edge printed composite. For the strength data, the retrieved back-calculated constants for use in Equations (8) and (13) are presented in Table 6.

Those equations permit the calculation of the longitudinal and transversal tension strength for a given volumetric fiber fraction. However, there are limits to the practical volumetric fiber fraction of the manufacturing method, with FFF AM of composite thermoplastic matrices employed, typically with an upper bound of 40% [22]. Furthermore, the strength and the stiffness of a composite can be influenced by tabbing and grips of the testing equipment. Wisnom et al. [55,56] studied this effect extensively for traditional manufacturing composites, while Pyl et al. [39] show the influence of the architecture and gripping system on the stiffness and strength determination for FRAM.

### 5.3. Machine Learning Output

**Figure 7:**
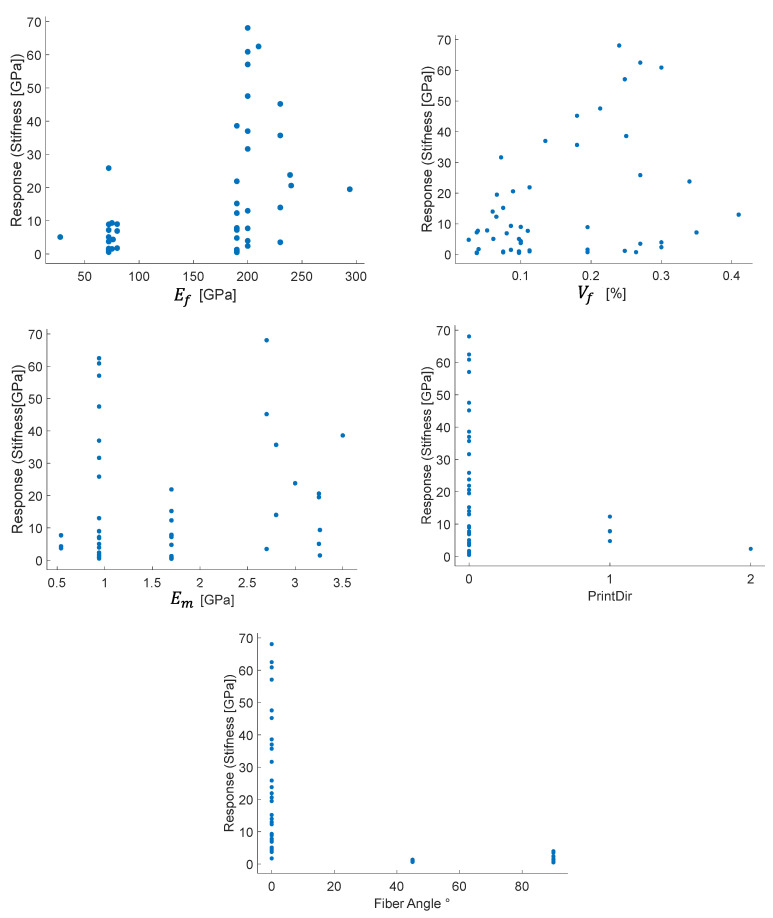
Response plots of the stiffness for five factors of the FRAM.

In analyzing data, the response plots for the different factors help identify visual patterns, filter data or identify outlier points. For example, Figure 7 shows the response plot for the machine learning data. Clusters are identifiable in the $E_{f}$, consistent with the discrete nature of fiber types, in the $E_{m}$ with matrix types, and the printing direction. From this response plot, it is not possible to identify a single trend; this is due to the large variation of the parameters and the interactive effect of variables. For instance, if tested at a transversal angle (90°), low carbon-fiber AM could give response values similar to fiberglass in longitudinal tension (0°).

The models were evaluated according to four performance metrics: root mean square error (RMSE), the coefficient of determination R-squared, the prediction speed, and the training time. R-squared is a number always smaller than one and usually larger than zero, comparing the constant average model with the trained model. Thus, the R-squared is negative if the predicting model is worse than a constant model. Table 7 resumes the model performance for the stiffness and strength response.

**Figure 8:**
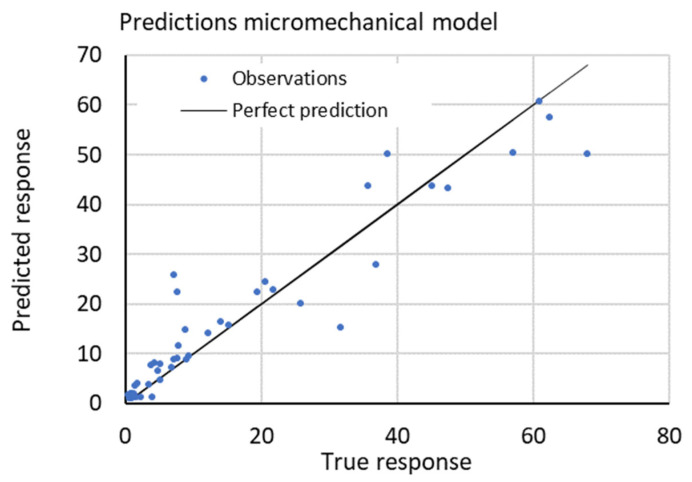
Predicted values of stiffness vs. true response.

**Figure 9:**
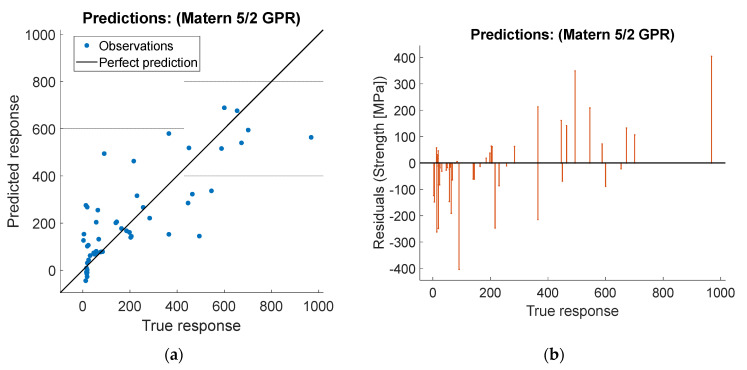
(**a**) Predicted values of strength vs. true response and (**b**) residuals of stiffness vs. true response.

Notice the better performance of the micromechanical-based method from the R-Squared column, followed by the 5/2 Matern Gaussian process regression. The fastest models in prediction capabilities are the linear regression with 630 milliseconds and the rational quadratic gaussian with 670 milliseconds. However, they are not the fastest to train, those are the Gaussian SVM and the Matern 5/2 GPR with 1.55 and 1.56 s, respectively. In contrast, the neural networks models did not capture the stiffness or strength response accurately, the best amongst them was the trilayered NN, and deeper NN could be employed to enhance the prediction capabilities. Kernel-based (Matern 5/2) and gaussian-based models (Gaussian SVM and GPR) showed the best performance. In comparing the micromechanics model with the plots of the data-driven winning model, Figure 8 and Figure 9 show a more negligible scattering of experimental points in the micromechanical model is inferred.

### 5.4. Failure Analysis and Microstructural Description

**Figure 10:**
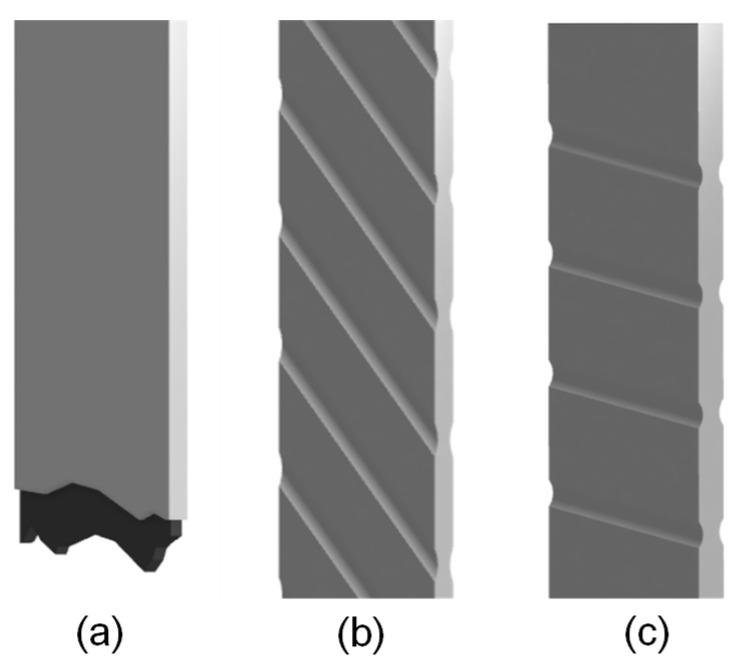
Failure topologies of AM Composites: (**a**) 0° fiber alignment, (**b**) 45°, (**c**) 90°.

The macroscopic appearance of failed specimens depends on processing parameters, such as volumetric fiber content and printing direction. Failure topologies could be resumed in three categories for the flat specimens, as shown in Figure 10. In the first type depicted in Figure 10a, a zero-degree tension failure is represented. All zero degrees coupons failed translaminar, meaning a breakage of the fibers and splitting the specimen into two parts.

In the second and third types, Figure 10b,c, for types 45° and 90°, respectively, the specimens did not break into separate pieces, and they suffered extensive deformation that reached the end of the experiment. The failure sequence may be that the failure starts in an intralaminar manner (through the thickness in which only matrix and fiber/matrix interface are broken) and then, interlaminar. In multiple points of the reinforced region, the damage progresses through the nylon, causing plastic deformation with high strains, thus, forming shear bands.

We saw a mixture of the two modes for the on-edge printing specimens. In specimens with low reinforcement content, the samples failed mainly by large deformation in the nylon zone and, consequently, the intralaminar failure of the reinforced region. On the other side of the spectrum, the specimens with higher reinforcement content failed by breaking the specimen into two pieces, caused by the reinforced region translaminar failure and the inability of the nylon to sustain the applied displacement.

**Figure 11:**
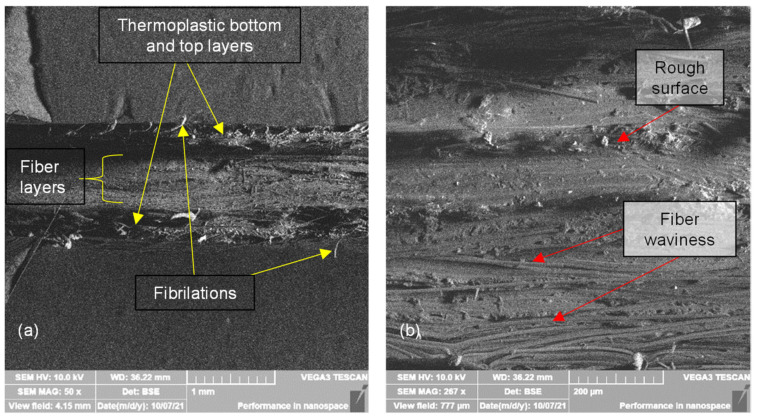
SEM transversal cut of a 55/110 layers carbon fiber on-edge printed sample: (**a**) 50×, (**b**) 267×.

Fractured specimens are dissected near the failure surface with a continuous saw using slow cutting rates. Cross-sectional views are from failed specimens ground in increasing grit paper numbers from 150 to 1500 grits, then stored in a dissector. Thermoplastic composite preparation is a delicate issue despite the care taken because abrasive particles could cause torn and rough surfaces [57], as shown in in Figure 11b. Figure 11 also shows in-plane fiber misalignment and low severity waviness. SEM micrographs are taken in a Quanta FEG 650 and a Vega Tecscan.

**Figure 12:**
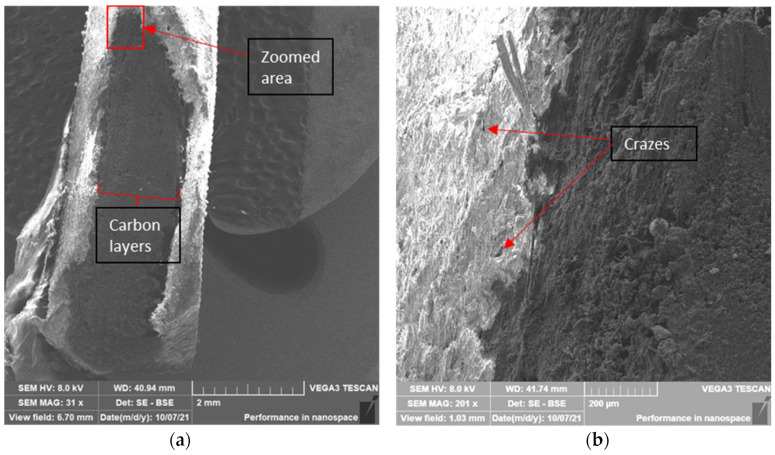
SEM transversal cut of a 55/110 layers carbon fiber on-edge printed sample: (**a**) 31×, (**b**) 201×.

The on-edge printing specimens show extensive damage in the nylon region, which can be concluded from the fibrillations in Figure 11a. In addition, other fractographic features such as scarps and crazes are visible in the nylon region in Figure 12a,b.

**Figure 13:**
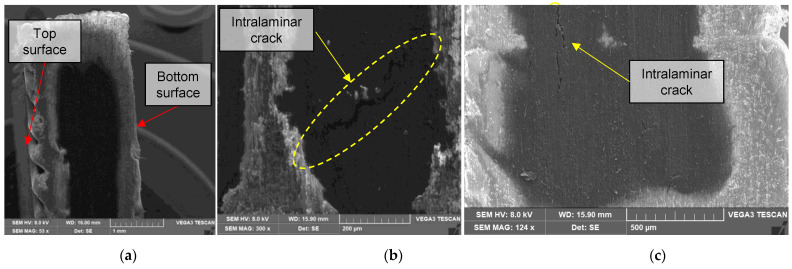
SEM transversal cut of a 4/16 carbon layers 45° flat printed sample: (**a**) 53×, (**b**) 300×, (**c**) 124×.

It is interesting to note the microstructural mismatch between the nylon region present at the bottom of the sample and the top surface for the flat specimens. This difference in surfaces is due to a lack of compaction phase in the last layers of the additive manufacturing process, thus, creating a rougher surface in which the raster print is more visible at the top (Figure 13a). Intralaminar failure is the most common failure present in these specimens. Figure 13b shows a crack in the reinforced region and Figure 13c in the vertical direction. The local stress field influences the path of the propagating crack, causing it to deviate.

**Figure 14:**
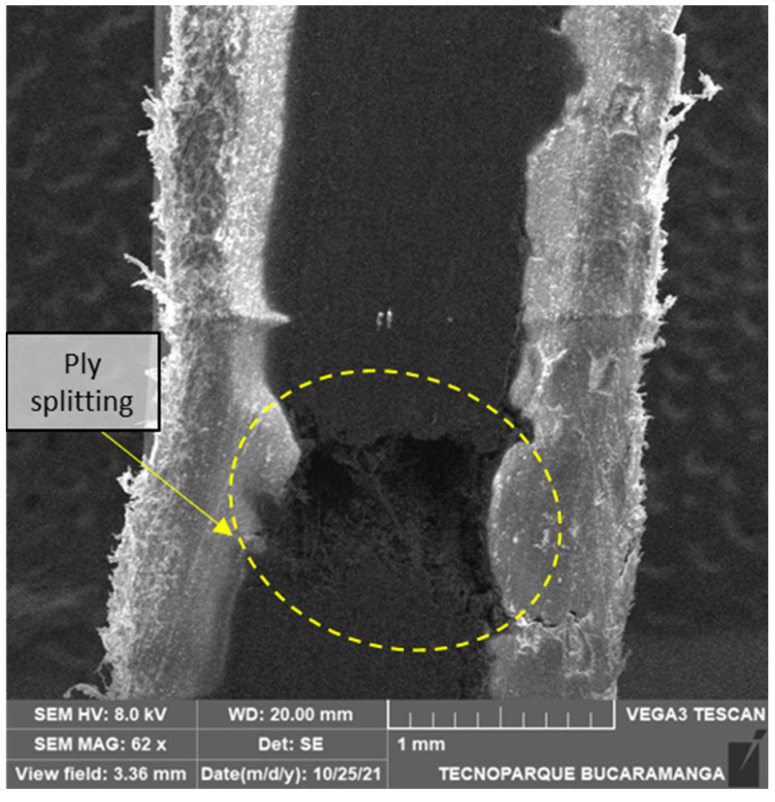
SEM transversal cut of a 6/16 carbon layers 45° flat printed sample.

**Figure 15:**
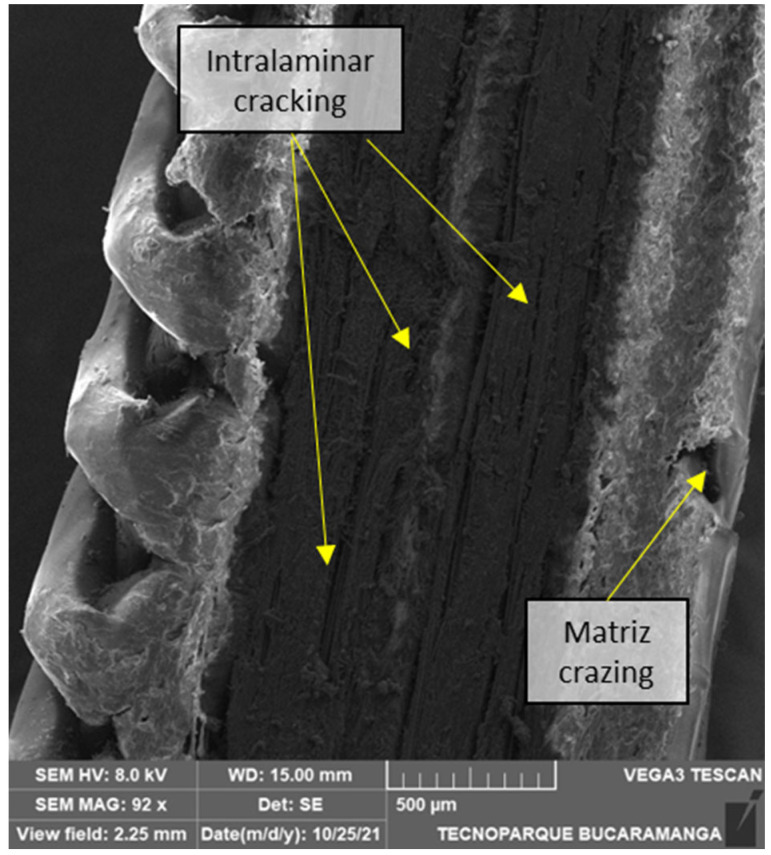
SEM transversal cut of a 4/16 carbon layers 90° flat printed sample.

The specimen in Figure 14 may have failed under intralaminar fracture followed by interlaminar, as the detachment of the reinforced plies and the nylon indicates. Ply splitting is one of the most common failure modes in laminated composites, sometimes called matrix cracking. This fracture mode develops from tensile forces transverse to the fibers or shear forces parallel to the fibers. In Figure 15, we can observe the extensive crack growth in a 90° sample consistent with an intralaminar failure and the crazes in the nylon bottom part.

**Figure 16:**
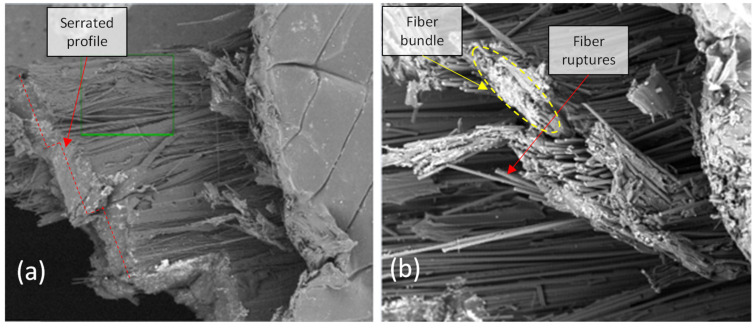
SEM fractography of a tensile test for a 90° reinforced specimen. (**a**) 100× magnification, (**b**) 400× magnification.

In Figure 15, the fracture morphology was consistent with an intralaminar failure mode. Due to its high toughness compared with other polymeric matrix systems, nylon presents a rough surface in which the fracture is principally absorbed through void coalescence, so large-scale ductile drawn and fibrillation occurs [57]. In addition, at slow speeds, such as those presented in this test, the matrix has time for plastic deformation, and fibrillation of the matrix develops. In Figure 16a, a serrated profile with planar surfaces is observable, while Figure 16b shows bundles of fibers that failed in the same plane. In 90° specimens, not all fiber sections are transverse to the load. Fibers make U-turns at the edges, as it is printed continuously, making them more susceptible to longitudinal tensile failure at the corners.

**Figure 17:**
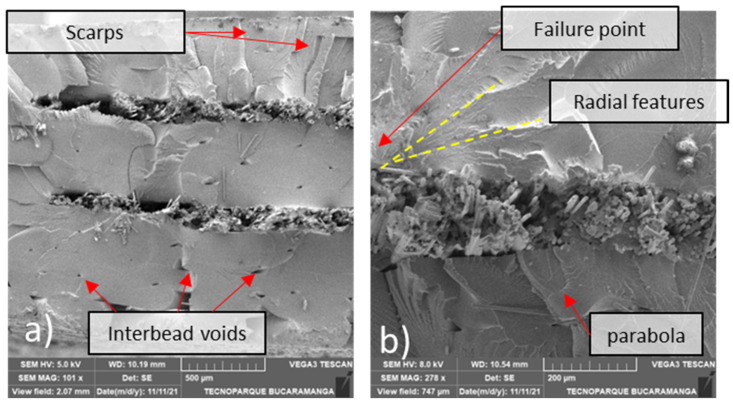
Flat 0° 4/16. (**a**) Wide view at 161× (**b**) Zoomed at fiber region.

**Figure 18:**
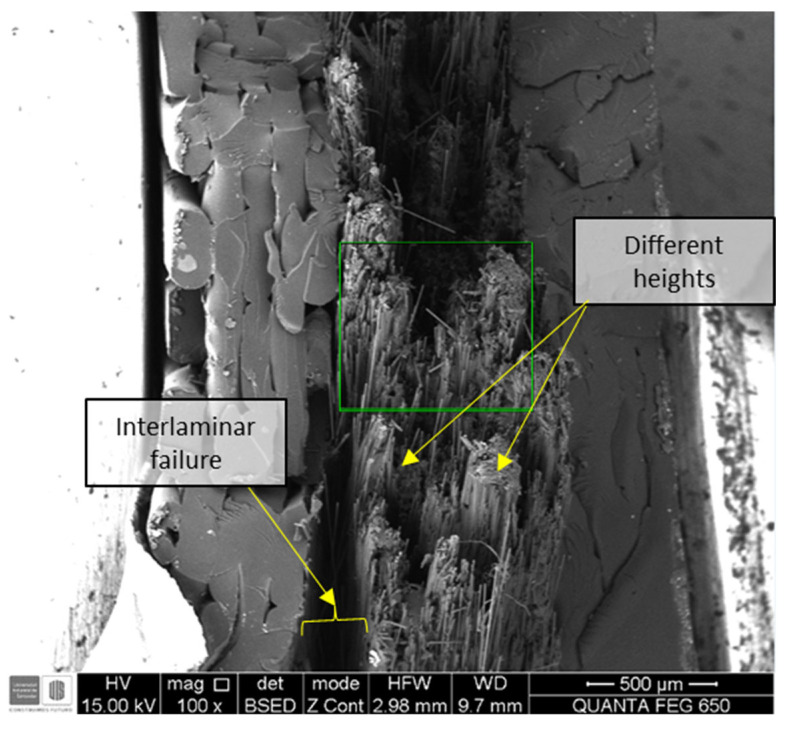
Fractography of a 0° 6/16 fiber-reinforced tensile test.

For the zero degrees flat printed specimen, the macroscopic tension appearance is shown in Figure 17a and Figure 18. Failure initiates at the location of small defects. The surface is relatively flat at the points close to it. All the fibers in this zone fractured in the same plane, parabolas in the nylon, and features that spread out of the possible failure zone are present. The fibers in different heights are consistent with a high-energy fracture.

Figure 18 shows a more significant amount of fiber debonding on the surface than Figure 17 Thermoplastic matrices often exhibit increased fiber pullout, leading to fewer bundles of failed fibers and “directly attributable fiber failure” (DAFFS).

**Figure 19:**
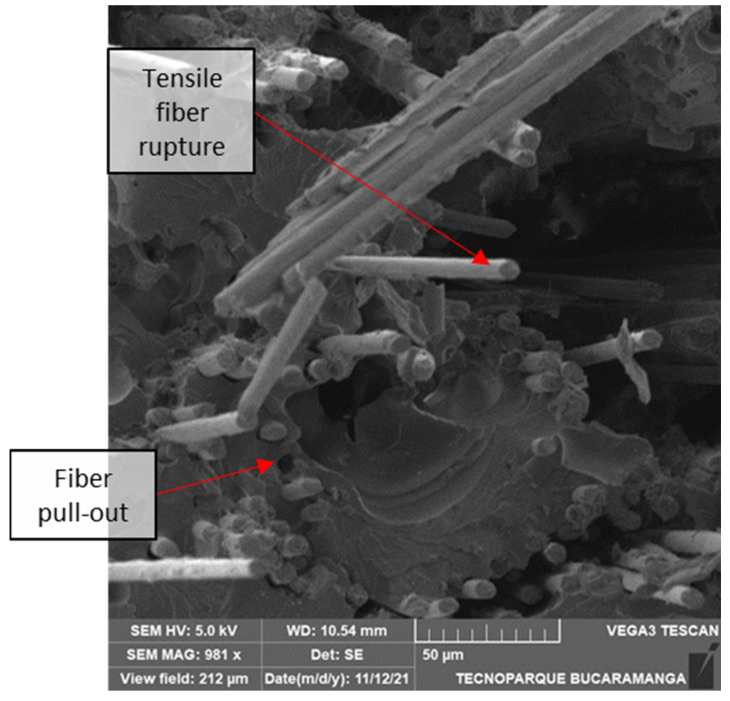
Flat 0° 2/16-11.

For the microscopic features of unidirectional tension, in Figure 19, the fiber ends exhibit a planar surface with a relatively perpendicular failure plane, indicating brittle longitudinal failure. Moreover, fiber pullout voids are visible, indicating low matrix attachment and, thus, poor fiber-matrix interphase strength.

## 6. Conclusions

The effect of the volumetric fiber fraction and printing direction on the mechanical response and failure mechanisms of additive manufacturing composites was assessed. This work tested different models to evaluate stiffness and strength prediction capabilities. Micromechanical models outperformed machine learning with an RMSE of 7.66 GPa in stiffness and 70.05 MPa in strength. In addition, they have the advantage of being physics-based. However, the performance of the ML algorithms was not very good in part due to a lack of consistent data, with a widespread range of materials, methods, and machines for the production of continuous FRAM. Poor data amount derives from the difficulty in obtaining data, and the lack of a standard for reporting the printing process and parameters. For instance, the articles did not report most of the assumed flat printing direction. Moreover, volumetric fiber fraction is not always reported. Among the ANNs, the trilayered NN performed the best.

Considerable strength variations were observed, and there are many reasons for this significant difference. First, is a lack of consistent definition of strength: the maximum stress or the linear elastic stress, also known as yield. Moreover, intrinsic variability and the effect of the manufacturing process means the provided materials could have a microporous difference in the reinforcing fibers. The manufacturing process feed rate, humidity, and the adhesion of the part into the building plate are variables that modify the strength [58,59]. Moreover, poor test methods impact the results and, although this effect was minimized with careful manipulation of the specimens, it was not possible to ensure the same conditions in other experimental work. Such conditions include the use and type of grip tabs. The results of stiffness and strength were independent of the stack-up order (this holds for longitudinal testing, such as tension and compression). However, a flexural model will give inaccurate results. One could then express the individual compliance matrices of the laminate.

Failure mechanisms, macroscopic damage appearance, and microstructural differences in specimens were observed. In specimens where fibers were aligned to zero degrees, there is a tendency to fail in a translaminar manner. At the same time, the other tested angles exhibited extensive intralaminar damage and plastic drawn out of the matrix.

The generalization capabilities of machine learning algorithms are good, giving reasonable estimates of the longitudinal and transverse modulus and strength of AM composites. However, the lack of an estimate of the other directions’ properties makes its generalization relatively poor. A possible way to overcome this issue would be to perform a data augmentation based on micromechanical formulations, preferably more accurate ones. Thus, the two models will cooperate instead of competing.

The model assumes a perfect bonding between layers, which in AM components is difficult to obtain. In addition, defects, such as bed level issues, thermal management of the extruder, warping of the piece, and hygroscopic characteristics of the nylon, can affect the interlayer bonding. Although, in reality, we have a composite sandwich in which forces are applied to a laminate, thus, this work aimed at comparing a basic model in which the mesoscale is not entirely depicted and instead gives an estimate of the mechanical properties.

## Figures and Tables

**Table 1:** DOE information using three factors.

Print Direction	Fiber Fraction	Fiber Angle	Total Tests
Flat	3 levels (3.75%, 7.50%, 11.25%)	0°, 45°, 90°	9
On-edge	4 (2.60%, 3.90%, 5.22%, 6.53%)	0°	4
		Total	13 × 4 replica

**Table 2:** Results for continuous carbon fiber reinforced Nylon White.

Reinforced Layers—Total Layers	$V_{f}$	Fiber Angle	Young’s Modulus (MPa)	COV (%)	Max. Stress (MPa)	COV (%)
	Carbon fiber flat					
2–16	3.75%	0°	7232.5	6.7	77.40	38.7
4–16	7.50%	0°	15204.0	0.5	198.83	11.9
6–16	11.25%	0°	21896.5	12.7	209.35	29.2
2–16	3.75%	45°	663.0	0.5	18.44	0.5
4–16	7.50%	45°	969.6	14.2	19.15	4.0
6–16	11.25%	45°	1789.0	24.3	19.08	18.4
2–16	3.75%	90°	503.5	8.3	16.67	4.0
4–16	7.50%	90°	727.3	19.3	15.39	1.8
6–16	11.25%	90°	1052.3	7.9	15.85	9.9
	Carbon fiber on-edge					
28–110	2.60%	0°	4893.3	1.7	47.61	27.4
42–110	3.90%	0°	7738.8	36.9	46.30	17.7
55–110	5.22%	0°	7855.8	38.1	57.14	25.7
70–110	6.53%	0°	13258.0	6.4	63.00	39.3

**Table 3:** Longitudinal and transversal modulus comparison: experimental and micromechanics formulation for various samples.

Coupon Info	Fiber Fraction [%]	$E_{1}(\mathrm{VAS})\;\mathrm{Model}\;(\mathrm{GPa})$	E1 (Exp.) (GPa)	Relative Error (%)	$E_{2}(\mathrm{VAS})\;\mathrm{Model}\;(\mathrm{GPa})$	E2 (Exp.) (GPa)	Source
cCf-PA	3.8%	8.60	7.23	16%	1.77	0.50	This work
cCf-PA	7.5%	15.67	15.20	3%	1.84	0.73	This work
cCf-PA	11.3%	22.73	21.90	4%	1.91	0.99	This work
cCf-PA ^a^	2.60%	6.856	4.89	29%	1.74	NA	This work
cCf-PA ^a^	3.90%	9.434	7.74	18%	1.77	NA	This work
cCf-PA ^a^	5.22%	12.05	7.86	35%	1.79	NA	This work
cCf-PA ^a^	6.53%	14.65	13.26	9%	1.82	NA	This work
cCf-PLA	6.6%	22.44	19.50	13%	3.48	NA	[4]
cJute-PLA	6.1%	4.72	5.11	−8%	3.43	NA	[40]
cCf-PA	6.0%	15.02	14.00	7%	2.98	NA	[41]
cCf-PA	18.0%	42.46	35.70	16%	3.41	NA	[41]
cCf-PLA	34.0%	80.05	23.80	70%	4.22	NA	[42]
cKv-PA	4.0%	4.09	1.77	57%	1.00	NA	[43]
cKv-PA	8.0%	7.25	6.92	5%	1.02	NA	[43]
cKv-PA	10.0%	8.83	9.02	−2%	1.04	NA	[43]
cCf-PLA	8.9%	24.30	20.60	15%	4.6	NA	[44]
cAramid-PLA	8.6%	9.52	9.34	2%	3.55	1.53	[45]
cCf-PA	27.0%	63.34	62.50	1%	4.44	NA	[46]
cCf-PA	18.0%	42.79	45.20	−6%	3.00	NA	[47]
cCf-PA	27.0%	63.34	NA	NA	2.32	3.53	[47]
cCf-PLA	25.0%	60.12	38.60	36%	4.64	NA	[48]
cCf-PA	24.0%	73.29	68.08	7%	2.32	1.22	[14]
cFg-PA	27.0%	21.76	25.86	−18%	1.28	1.22	[14]
cCf-PA	13.5%	41.97	37.00	12%	1.96	NA	[4]
cCf-PA	41.0%	98.85	13.00	87%	1.56	NA	[49]
cCf-PA	35.0%	81.10	7.20	91%	1.44	NA	[49]
cCf-PA	30.0%	61.19	60.90	0%	2.42	3.97	[50]
cCf-PA ^b^	30.0%	2.42	2.40	1%	NA	NA	[50]
cCf-PA	21.3%	50.33	47.56	5%	2.16	NA	[39]
cCf-PA	24.8%	58.32	57.09	2%	2.26	NA	[39]
cCf-PA	7.2%	18.14	31.65	−74%	1.83	NA	[39]
cFg-PA	9.8%	7.94	5.09	36%	1.04	0.58	[51]
CFg-PA	19.5%	14.87	8.92	40%	1.16	1.61	[51]
cCf-PA	11.0%	22.84	7.73	66%	1.06	NA	[7]
cKv-PA	10.0%	8.45	4.37	48%	1.04	NA	[7]
cFg-PA	10.0%	8.05	3.75	53%	1.04	NA	[7]

Notes: ^a^: On-edge printed, ^b^. upright printed.

**Table 4:** Suggested values of the engineering constants in continuous FRAM composites.

	$V_{f}(\%)$	$E_{3}(\mathrm{GPa})$	$v_{12}$	$v_{13}$	$G_{12}(\mathrm{GPa})$	$G_{13}(\mathrm{GPa})$	$G_{23}(\mathrm{GPa})$
Ccf-PA	27	2.32	0.336	0.336	1.24	1.24	1.15
Cfg-PA	27	2.31	0.336	0.336	1.22	1.22	1.13
Ckv-PA	10	1.88	0.351	0.351	0.87	0.87	0.846
cCf-PLA	9	3.84	0.352	0.352	1.77	1.77	1.717
cJute-PLA	6	3.45	0.35	0.352	1.32	1.32	1.28

**Table 5:** Processed data for strength comparison for FRAM.

	Flat Printing Direction				
Reinforced Layers—Total Layers	Fiber Angle	$V_{f}$(%)	Max Stress Exp. (MPa)	Predicted (MPa)	Error (%)
2–16	0°	3.75	77.40	92.34	19.30
4–16	0°	7.50	198.83	171.47	13.76
6–16	0°	11.25	209.35	250.59	19.70
2–16	90°	3.75	16.67	17.777	6.21
4–16	90°	7.50	15.39	15.952	3.50
6–16	90°	11.25	15.85	14.701	7.80
	On-edge printing direction				
28–110	0°	2.60	47.61	35.17	35.37
42–110	0°	3.90	46.30	49.34	6.16
55–110	0°	5.22	57.14	63.72	10.30
70–110	0°	6.53	63.00	78.00	19.20

**Table 6:** Retrieved back-calculated constants for continuous FRAM.

Material	$F_{fT}(\mathrm{MPa})$	$F_{mT}(\mathrm{MPa})$
Carbon-fiber Polyamide	2110; 1090 ^a^	34.55
Fiberglass Polyamide	1185	49.10
Kevlar-Polyamide	891	NA
Carbon-fiber PLA	1749	NA

Note: ^a^ for on-edge printed direction.

**Table 7:** Comparative table for the predicting model performance.

	Stiffness				Strength			
MODEL	RMSE (GPa)	R-Squared	Prediction Speed (ms)	Training Time [S]	RMSE (MPa)	R-Squared	Prediction Speed (ms)	Training Time (s)
Fine tree	16.859	0.19	780	3.76	167.53	0.55	2400	0.835
Linear regression	14.984	0.36	630	3.95	183.31	0.46	1600	0.9456
Linear SVM	16.245	0.24	1200	3.02	176.24	0.5	1600	1.2923
Gaussian SVM	13.475	0.48	1500	0.59	162.92	0.57	2200	0.8316
Rational quadratic Gaussian	11.064	0.65	670	8.61	146.72	0.65	1800	1.377
Matern 5/2 GPR	10.905	0.66	930	1.55	142.87	0.67	2500	1.2855
Exponential GPR	11.092	0.65	1200	1.56	140.21	0.68	1600	1.1625
Narrow Neural network	46.228	−5.12	1000	6.81	1014.8	−15.58	1800	5.042
Medium Neural network	30.128	−1.6	1600	3.60	445.59	−2.2	1700	3.99
Wide Neural Network	30.584	−1.68	1900	4.34	481.14	−2.73	2700	3.38
Trilayered Neural network	18.622	0.01	1900	5.09	302.25	−0.47	2600	4.13
Micromechanics	6.81	0.74	NA	NA	70.65 ^a^	0.72	NA	NA

## Data Availability

Data available on request due to restrictions in privacy. The data presented in this study are available on request from the corresponding author. The data are not publicly available due to confidentiality.

## Appendix A

**Table A1:** Experimental data obtained following ASTM D3039.

$E_{f}$ **(GPa)**	$S_{f}$ **(MPa)**	$V_{f}$	$E_{m}$ **(GPa)**	$S_{m}$ **(MPa)**	$\nu_{f}$	$\nu_{m}$	PrintDir	Fiber Angle (°)	Stiffness Output (GPa)	Strength Output (MPa)
294.0	5880	0.066	3.25	42.6	0.27	0.35	0	0	19.50	185.2
27.4	417	0.061	3.25	42.6	0.38	0.35	0	0	5.11	57.1
230.0	3530	0.060	2.80	50.0	0.27	0.39	0	0	14.00	140.0
230.0	3530	0.180	2.80	50.0	0.27	0.39	0	0	35.70	464.4
239.0	3965	0.340	3.00	28.0	0.27	0.35	0	0	23.80	91.0
79.8	3620	0.040	0.94	31.0	0.36	0.39	0	0	1.77	31.0
79.8	3620	0.080	0.94	31.0	0.36	0.39	0	0	6.92	60.0
79.8	3620	0.100	0.94	31.0	0.36	0.39	0	0	9.00	84.0
240.0	4100	0.089	3.25	42.6	0.27	0.35	0	0	20.60	256.0
75.0	3400	0.086	3.26	34.0	0.36	0.35	0	0	9.34	203.0
75.0	3400	0.086	3.26	34.0	0.36	0.35	0	90	1.53	3.0
190.0	3800	0.038	1.70	51.0	0.27	0.39	0	0	7.23	67.6
190.0	3800	0.075	1.70	51.0	0.27	0.39	0	0	15.20	198.0
190.0	3800	0.113	1.70	51.0	0.27	0.39	0	0	21.90	229.8
190.0	3800	0.038	1.70	51.0	0.27	0.39	0	45	0.66	18.4
190.0	3800	0.075	1.70	51.0	0.27	0.39	0	45	0.97	19.1
190.0	3800	0.113	1.70	51.0	0.27	0.39	0	45	1.34	19.2
190.0	3800	0.038	1.70	51.0	0.27	0.39	0	90	0.50	16.7
190.0	3800	0.075	1.70	51.0	0.27	0.39	0	90	0.73	15.4
190.0	3800	0.113	1.70	51.0	0.27	0.39	0	90	1.05	15.9
190.0	3800	0.026	1.70	51.0	0.27	0.39	1	0	4.81	50.1
190.0	3800	0.039	1.70	51.0	0.27	0.39	1	0	7.74	46.3
190.0	3800	0.052	1.70	51.0	0.27	0.39	1	0	7.86	57.1
190.0	3800	0.065	1.70	51.0	0.27	0.39	1	0	12.32	76.5
210.0	3800	0.270	0.94	31.0	0.27	0.39	0	0	62.50	968.0
230.0	3800	0.180	2.70	51.0	0.2	0.3	0	0	45.20	493.9
230.0	3800	0.270	2.70	51.0	0.2	0.3	0	90	3.53	13.5
190.0	3800	0.250	3.50	42.6	0.27	0.35	0	0	38.60	446.0
200.0	3800	0.240	2.70	31.0	0.27	0.39	0	0	68.08	588.0
72.0	3450	0.248	0.94	31.0	0.21	0.39	0	90	1.22	12.6
72.0	3450	0.270	0.94	31.0	0.21	0.39	0	0	25.86	545.4
72.0	3450	0.264	0.94	31.0	0.21	0.39	0	45	0.78	63.9
200.0	3800	0.135	0.94	31.0	0.27	0.39	0	0	37.00	365.0
200.0	3800	0.410	0.94	31.0	0.27	0.39	0	0	13.00	600.0
72.0	3450	0.350	0.94	31.0	0.21	0.39	0	0	7.20	450.0
200.0	3800	0.300	0.94	31.0	0.27	0.39	0	0	60.90	701.0
200.0	3800	0.300	0.94	31.0	0.27	0.39	0	90	3.97	19.0
200.0	3800	0.300	0.94	31.0	0.27	0.39	2	90	2.40	5.1
200.0	3800	0.213	0.94	57.0	0.27	0.39	0	0	47.56	672.5
200.0	3800	0.248	0.94	57.0	0.27	0.39	0	0	57.09	654.0
200.0	3800	0.072	0.94	57.0	0.27	0.39	0	0	31.65	365.0
72.0	3450	0.098	0.94	54.0	0.21	0.39	0	0	5.09	143.6
72.0	3450	0.098	0.94	54.0	0.21	0.39	0	45	1.02	24.8
72.0	3450	0.098	0.94	54.0	0.21	0.39	0	90	0.58	18.3
72.0	3450	0.195	0.94	54.0	0.21	0.39	0	0	8.92	283.5
72.0	3450	0.195	0.94	54.0	0.21	0.39	0	45	0.79	23.4
72.0	3450	0.195	0.94	54.0	0.21	0.39	0	90	1.61	18.4
200.0	3800	0.110	0.54	61.0	0.27	0.39	0	0	7.73	216.0
76.0	3620	0.100	0.54	61.0	0.36	0.39	0	0	4.37	164.0
72.0	3450	0.100	0.54	61.0	0.21	0.39	0	0	3.75	206.0
